# Oral lichenoid lesions associated with amalgam restorations: A prospective pilot study addressing the adult population of the Basque Country

**DOI:** 10.4317/medoral.17733

**Published:** 2012-02-09

**Authors:** Maria J. Lartitegui-Sebastián, Begoña Martínez-Revilla, Carolina Saiz-Garcia, Sonia Eguizabal-Saracho, Jose M. Aguirre-Urizar

**Affiliations:** 1 Associate Prof of Dental Pathology and Therapeutics. Unit of Oral Medicine and Master in Oral Pathology. Clinical Odontology Service and Department of Stomatology. University of the Basque Country, Faculty Medicine and Dentistry; 2 Postgraduate Dental Students, Master in Oral Pathol. Unit of Oral Medicine and Master in Oral Pathology. Clinical Odontology Service and Department of Stomatology. University of the Basque Country, Faculty Medicine and Dentistry; 3 Postgraduate Dental Students Unit of Oral Medicine and Master in Oral Pathology. Clinical Odontology Service and Department of Stomatology. University of the Basque Country, Faculty Medicine and Dentistry; 4 Professor of Oral Medicine. Unit of Oral Medicine and Master in Oral Pathology. Clinical Odontology Service and Department of Stomatology. University of the Basque Country, Faculty Medicine and Dentistry

## Abstract

Oral lichenoid lesions (OLLs) are linked to a heterogeneous group of pathologies involving the oral mucosa that cannot be distinguished from the oral lichen planus excepting the fact that direct causal factors such as silver amalgam restorations (SARs) can be allocated to them. Purpose: To analyze the prevalence of mucosal lesions associated with SAR in a group of SAR carrying patients in the Basque Country. Study Design: A clinical prospective study was carried out on 100 adult patients over 30 years of age at the UPV/EHU Clinical Odontology Service whose rear teeth had at least one SAR. Patients were identified and mucosal lesions and amalgam restorations were characterized. Patch tests were performed on patients with lesions and amalgams were replaced with composite material. A statistical and comparative analysis was performed with the resulting data. Results: OLLs were found in 7 patients whose predominant lesion was bilateral, asymmetrical and asymptomatic white papule-macule. Lesions were related to old and corroded SARs. Patch testing was positive in two cases. SAR substitution produced an improvement in 5 cases. Conclusions: The presence of lichenoid lesions associated with SARs is infrequent in our environment and is preferentially related to old and corroded restorations.

** Key words:**Oral mucosa, lichenoid lesions, restoration, silver amalgam, patch test.

## Introduction

Dental materials can produce allergic contact reactions in the mouth with an extensive clinical presentation (1-4).

Silver amalgams have been frequently used in dentistry and can produce hypersensitivity lesions in the oral mucosa in the form of an oral lichenoid lesion (OLL) (5-12).

OLLs form part of a heterogeneous group of chronic inflammatory diseases that are indistinguishable from the oral lichen planus (OLP) and produced by a type IV deferred hypersensitivity reaction triggered by extensive exposure to different antigens such as those pertaining to an amalgam (3,5,13). Mercury is the component most frequently associated with amalgam together with copper, zinc and tin, although to a lesser extent (1,10,14).

Amalgam-associated OLLs (AAgOLL) usually appear clinically in the form of white reticular papular lesions involving the mucosa occasionally with plaques and erosive, atrophic or ulcerated areas. Contrary to the OLP, these lesions are usually unilateral and/or asymmetrical and adjacent to amalgam restorations (14-16).

In terms of diagnosis, these lesions are clinical-pathological. Patch testing has been used to check their association with amalgam in an attempt to demonstrate allergy to amalgam components as well as the existence of a favourable course subsequent to coating or replacing other related restorations (3,11). It has been suggested that amalgams should be replaced in those cases in which lesions are in direct contact with the latter and whenever patch testing is positive (1,5,6,8).

Although nowadays silver amalgams are used much less as a sealing material in the field of restorative dentistry, many adults are still wearing restorations made of this material. In Spain, very few trials have been carried out to establish a connection between the presence of this material and the existence of lesions involving the oral mucosa (9).

The purpose of this paper is to address the prevalence of mucosal lesions associated with SARs in a group of patients with SARs in the Basque Country.

## Material and Methods

We have studied 100 consecutive patients at the Clinical Dentistry Service of the University of the Basque Country /EHU. The study was approved by the Research Ethics Committee of the University of the Basque Country /EHU (CEISH) and all patients gave their consent.

Inclusion criteria required that all patients be over 30 years old and with at least one silver amalgam restoration in their rear teeth.

The group studied consisted of 47 women and 53 males with an average age of 59 and whose minimum and maximum ages were 30 and 77 years respectively.

Lichenoid lesions of the oral mucosa were identified and qualified according to previously established criteria (17). A specific protocol was designed to analyze the location, physical appearance, symmetry and bilateral character of the lesions and their topographic relationship with silver amalgam restorations. The number, location, condition and colour of the surface (smooth/rough, silver/blackish) were assessed in the restorations.

Patch tests were performed on all patients with lichenoid lesions as established in the Torgersen et al. protocol (4). The allergen battery was designed specifically in relation to the amalgam (Martí Tor-I, S L. Madrid, Spain y Chemotechnique Diagnostics. Vellinge, Sweden) ( Table 1).

**Table 1:**
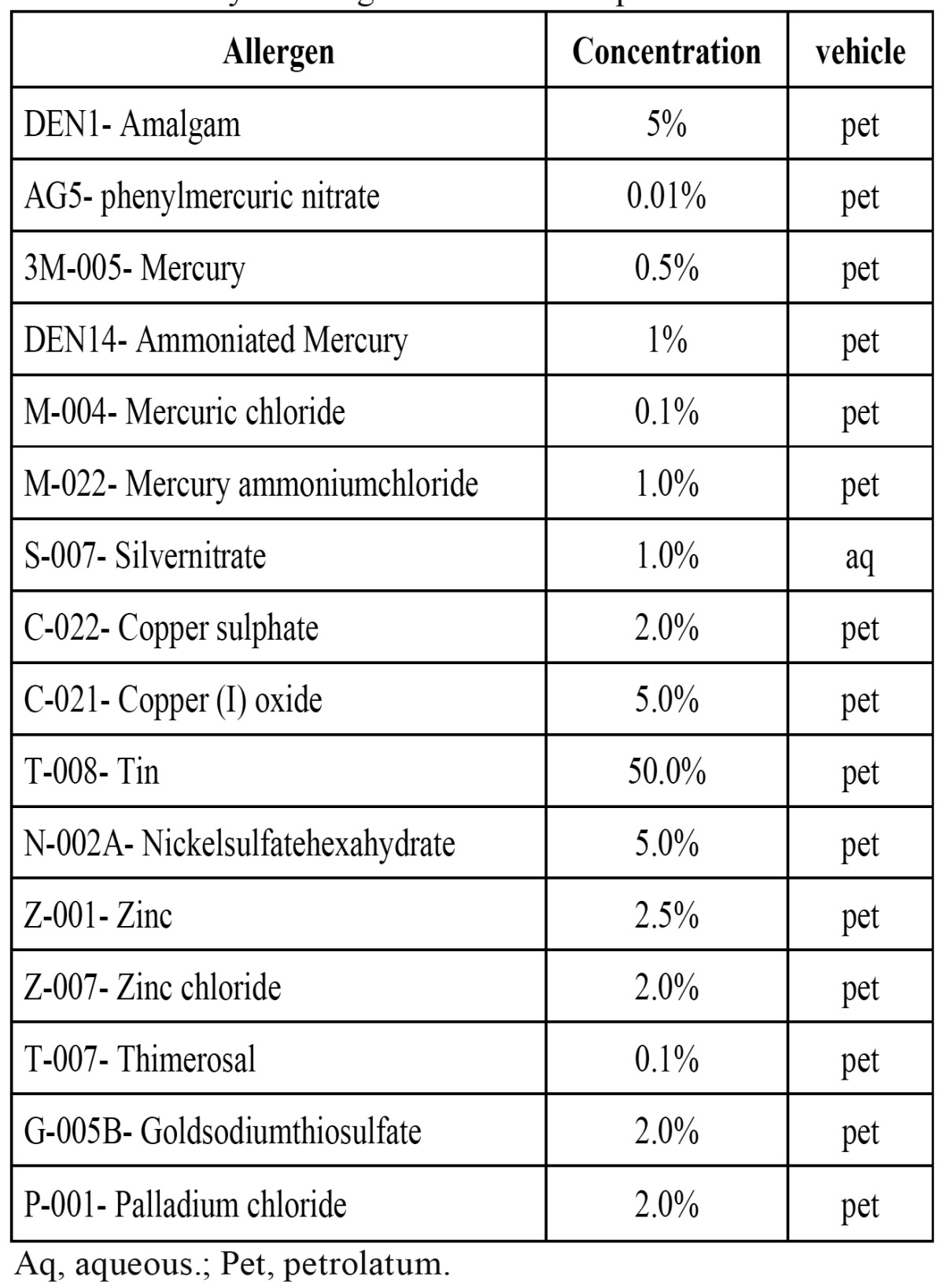
Battery of allergens tested in the patch test.

Amalgams were replaced with composite (Spectrum, Dentsply De Trey. Germany) in all patients with lichenoid lesions and were subsequently monitored at 3 and 6 months.

A statistic descriptive and comparative analysis was performed on data obtained with SPSS v 18.0 software (SPSS Inc. Chicago, IL, USA).

## Results

We found lichenoid lesions involving the mucosa in 7 patients. ( Table 2) shows most relevant patient data with and without lichenoid lesions.

**Table 2:**
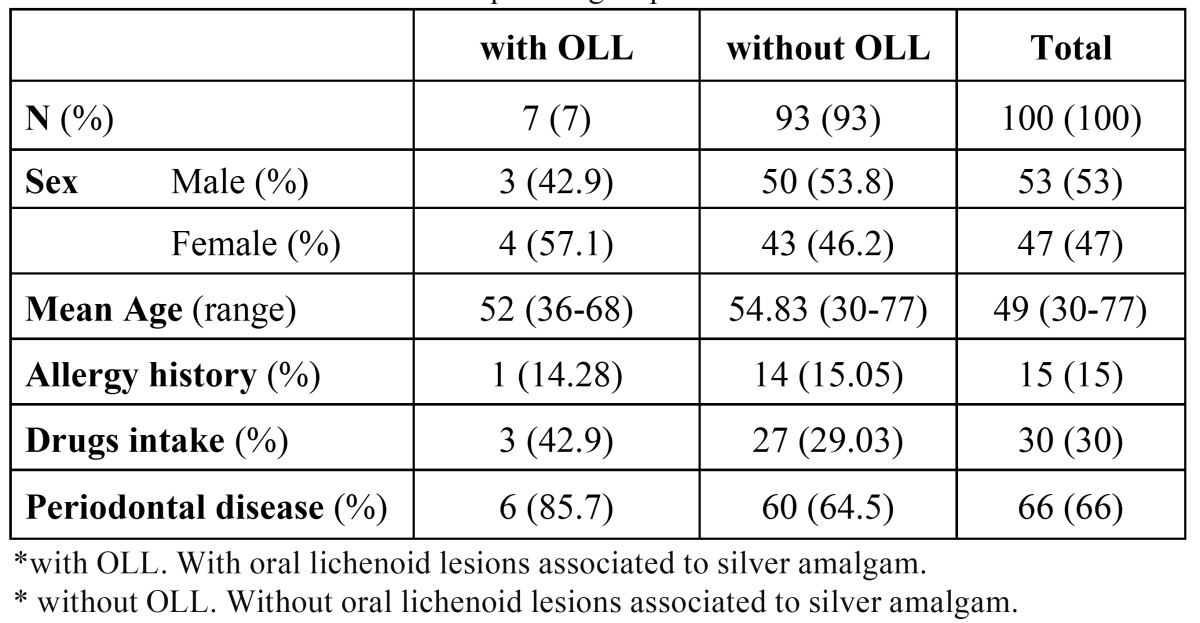
Characteristics of different patient groups.

In all cases, white maculas and papulas were the predominant mucosal lesions although erosive-ulcerative lesions were reported in only one case. Most lesions were bilateral and symmetrical (85.72%) and were found more frequently in the buccal mucosa (6 cases) followed by the lingual mucosa (2 cases). Only one case, however, featured a buccal and lingual location. Lesions were asymptomatic in six cases (85.72%) and pain was only reported in the case presenting erosive-ulcerative lesions.

Of the 7 patients, only one had an allergic disease (allergic to pollen); 3 patients were taking medication continuously although in no case was the onset of OLL (18) related to any drug. Six of the patients showed signs of a mild or moderate adult periodontal disease.

Observed OLLs were near AAg restorations in all instances. In 2 cases (28.6%) lesions were in direct contact, whereas in all other cases (71.4%) they were only adjacent and apparently not in direct contact with restorations.

In patients with OLL, the average number of teeth sealed with amalgam was five, with a minimum of 2 and a maximum of 7. The average number of surfaces sealed with silver amalgam was 7, with a minimum of 3 and a maximum of 12. On average, silver amalgams had been in the mouth for 27 years, 20 years in the case of the most recent placements and 40 in the case of the oldest. The surface colour of restorations was silver in 28.57% of the cases reported and blackish in 71.43%. Restorations were rough in 71.43% of the cases and smooth in 28.57% ( Table 3).

**Table 3:**
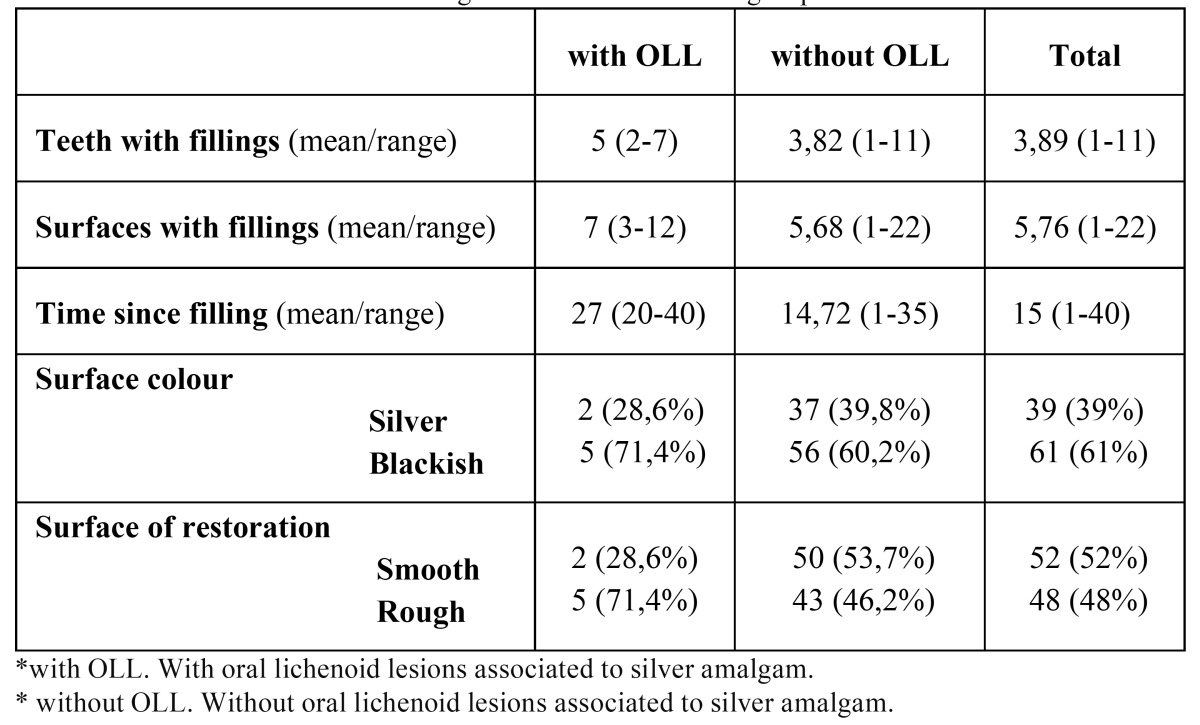
Characteristics of silver amalgam restorations in each group.

**Figure 1:**
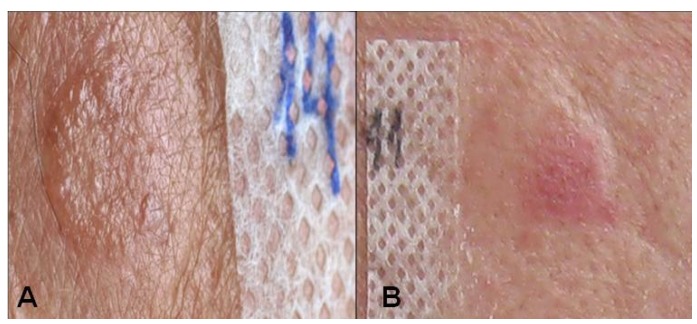
Positive patch test reactions: A: Thimerosal, B: Nickel.

Patch testing was only positive in 2 patients (28.57%); positive results were obtained with thimerosal (an organic component of mercury) and nickel (Fig. 1). No correspondence was found between these cases and those presenting direct contact with the amalgam.

**Figure 2:**
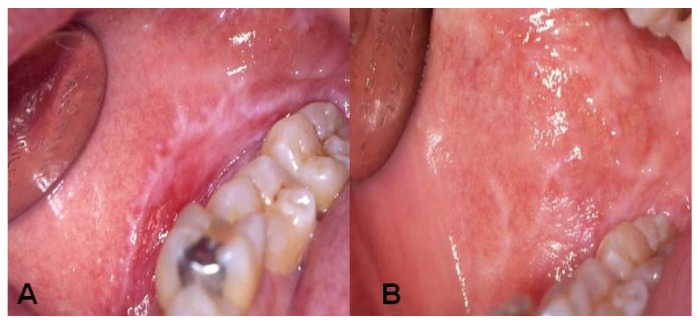
Evolution of the lesions after SAR replacement. A: Before amalgam removal, B: Three months after.

In all cases in which mucosal lesions were reported amalgams were replaced with composite restorations. Improvements were observed 3 months after replacing the amalgams with an amelioration of lesions in 5 (71.4%) patients, (Fig. 2). At six months, all lesions disappeared in 1 case although white residual papules persisted in all other cases.

## Discussion

It seems that lichenoid lesions associated with the presence of silver amalgams do not represent a pathology frequently found in our environment. It is difficult, however, to draw valid conclusions with regard to their true prevalence as no similar studies are available for comparison with our results. Most of the papers addressing the prevalence of these processes do so in a general manner by bringing all cases together under the generic diagnosis of lichen planus (5,13). A majority of the papers dealing with associated lichenoid lesions address the presence or absence of amalgam restorations in patients with “lichenoid lesions” (5,9).

Contrary to what other authors have stated in the past, we have not noted a marked bias towards the female gender, although our results do coincide in terms of age of onset (5-9,19).

As a rule, it has been stated that LLOAAg appear in the form of asymptomatic, unilateral or asymmetric white reticular papular lesions located in the yugal and lingual mucosa (11,13,15,16). Only one paper (19) says that erosive presentations are very commonplace in this process, although the clinical data supplied is somewhat misleading. In our study, reticular papulas have been reported as the predominant mucosal lesion together with the buccal and lingual mucosa as the most frequent locations. Nonetheless, and unlike the classical approach (13,15,16), most of the lesions found in our patients were bilateral and symmetric, a fact we have related to the bilateral presence of silver amalgams as described by Aggarwal et al. (12).

In general terms, and as noted in previous papers (2,11), lesions were asymptomatic and therefore unknown to the patient, with the exception of only one erosive case.

We believe that the disparities associated with the clinical features of these mucosal lesions are directly related to a lack of properly defined diagnostic clinical-pathological criteria. Our group believes that all processes that have in common white reticular papulae in the oral mucosa should be generically rated as “oral lichenoid diseases” for the purposes of establishing a more accurate diagnosis at a later stage (16,17). In most of the papers dealing with series comprising lichenoid lesions associated with silver amalgams it is probably true that a number of processes have been included: lichen planus, lichenoid lesions associated with drugs, idiopathic lichenoid lesions, erythroleukoplasia, etc. This would serve to explain the disparities found with regard to patch testing positivity in some studies and the fact that no improvements were obtained once the restorations had been removed (7,14).

It is very important to diagnose this pathology correctly as other authors have noted that the chances of triggering a malignant transformation are enhanced should a lichenoid lesion be involved instead of a lichen planus (20).

Although it is known that OLLAAgs are produced by a hypersensitivity phenomenon (3,5), we have only detected allergic reactivity in patch testing in less than one third of all the cases reported and which are in sharp contrast with those obtained by other authors (5,6) and range from 40 to 70% in terms of positivity. In these studies, however, patients associated with clinical data related to lichenoid lesions had been selected previously.

We believe that, as far as the condition of amalgam restorations is concerned, our results support a theory that suggests that these lesions will not only be caused by hypersensitivity to amalgam components, but also by other mechanisms. We would therefore be dealing with toxic-corrosive or even galvanic phenomena, although the latter have never been fully demonstrated (2). In this regard, our study has been able to ascertain that the average age of silver amalgam restorations in patients with lesions was very high, thus increasing the chances of producing corrosive damages affecting a rough and blackish surface. As suggested by some authors (5-8), metallic ions and corrosive products are released to unleash a toxic and irritating reaction in the adjacent oral mucosa that could produce lesions.

Replacing silver amalgams with an alternative material in all patients with lesions is a controversial issue. Although some authors are in favor of doing so in all instances, others only favor this option in those cases in which hypersensitivity has been established (14). Generally speaking, it has been noted that when dealing with a diagnosis of this kind, amalgams should be replaced in those cases in which the mucosal lesion is in direct contact and patch testing is positive (1,5,6,8). Other authors, however, (7,19) have noted that many patients with lesions do benefit from a replacement regardless of the outcome of the patch testing, thus supporting the hypothesis that states that lesions are produced by corrosive phenomena in many cases. Consequently, we believe it would be suitable to perform a replacement whenever a possible relationship with silver amalgams is established. Our results would support this view as improvements were reported in most patients and it was noted that lesions disappeared once silver amalgams were replaced.

Therefore, the following conclusions can be drawn from our work:

1.- That the presence of lichenoid lesions in patients with silver amalgam restorations is infrequent in our environment.

2.- That the lesions detected are mainly found in the buccal and lingual mucosa and are generally white, papular-macular and asymptomatic.

3.- That, in most instances, lesions are associated with the presence of old and corroded amalgams with a low level of hypersensitivity reactions.

4.- That the replacement of amalgam restorations produces significant improvements in most patients, regardless of patch testing outcomes.
